# MSC-induced lncRNA HCP5 drove fatty acid oxidation through miR-3619-5p/AMPK/PGC1α/CEBPB axis to promote stemness and chemo-resistance of gastric cancer

**DOI:** 10.1038/s41419-020-2426-z

**Published:** 2020-04-16

**Authors:** Honglei Wu, Bin Liu, Zhaosheng Chen, Guangchun Li, Zhen Zhang

**Affiliations:** Department of Gastroenterology, the Second Hospital of Shandong University, No.247 Beiyuan Street, 250033 Jinan, Shandong China

**Keywords:** Gastric cancer, Cell biology

## Abstract

Chemotherapy is the first-tier treatment regime for gastric cancer (GC) patients at advance stages. Mesenchymal stem cell (MSC) cam affect drug-resistance of GC cells in tumor microenvironment, but the detailed mechanism remains poorly understood. Present study aimed to investigate the regulation of MSC-induced long non-coding RNA (lncRNA) in GC. Dysregulated lncRNAs in GC were analyzed based on GEO data. Stemness and drug-resistance of GC cells were detected by sphere formation, colony formation, CCK-8, and flow cytometry analyses. MicroRNA (miRNA)-related pathways were analyzed by online KEGG analysis tool DAVID6.8. Molecular interactions were determined by luciferase reporter assay, pulldown, RNA immunoprecipitation (RIP), chromatin immunoprecipitation (ChIP), and co-immunoprecipitation (CoIP). Results revealed that MSC co-culture improved stemness and drug-resistance of GC cells. LncRNA histocompatibility leukocyte antigen complex P5 (HCP5) was induced in GC cells by MSC co-culture, contributing to stemness and drug-resistance. Mechanistically, HCP5 sequestered miR-3619-5p and upregulated PPARG coactivator 1 alpha (PPARGC1A), increasing transcription complex Peroxisome proliferator activated receptor (PPAR) coactivator‐1α (PGC1α)/CEBPB and transcriptionally inducing carnitine palmitoyltransferase 1 (CPT1), which prompted the fatty acid oxidation (FAO) in GC cells. In conclusion, MSC-induced lncRNA HCP5 drove FAO through miR-3619-5p/AMPK/PGC1α/CEBPB axis to promote stemness and chemo-resistance of GC, indicating that targeting HCP5 was a novel approach to enhancing the efficacy of chemotherapy in GC.

## Introduction

Gastric cancer (GC) has long been the uppermost cause of tumor-associated mortality^1,2^. Although operation‐oriented comprehensive therapy is considered as the primary choice for GC patients at advanced stages, the postsurgical 5‐year survival rate is merely around 20–50%^3^. Besides surgery, chemotherapy is the main clinical therapeutic tool against GC^4^. Unfortunately, resistance to drugs largely limits the efficacy of chemotherapy in GC^5^. Therefore, a better grasp of mechanism behind chemo-resistance in GC cells will help exploit new approaches to advancing treatment efficacy for GC patients.

Studies have attached great importance of tumor microenvironment to cancer cell resistance to drugs^6,7^. Of note, tumor microenvironment consists of diverse types of non-malignant cells, such as mesenchymal stromal cells (MSCs)^8^. Through secreting a series of cytokines, MSCs pose impacts on proliferation, metastasis, as well as angiogenesis of cancer cells^9,10^. Moreover, MSCs are demonstrated as contributors of tissue regeneration responding to therapy^11,12^. It is reported that MSCs help the acquisition of stem cell properties in cancer cells so that the chemo-resistance of cancer cells is better conferred^13–15^. Multiple studies have proved the strengthening effect of MSCs on chemo-resistance of tumor cells in vitro and in vivo^15–17^. Also, mounting works have depicted that MSCs are deeply involved in the development of tumor growth and drug resistance in GC^18,19^.

Dysregulated metabolism, recognized as the hallmark of cancer development^15^, is also involved in the mechanism of chemotherapy failure^20–22^. Fatty acid oxidation (FAO) is a major pathway regulating fatty acid degradation and promoting ATP and NADPH production^23,24^. Association between altered lipid metabolism mediated by FAO and tumor progression has been established^25,26^. Furthermore, FAO is delineated to support stem cell property and chemo-resistance of cancer cells^27^, and repression of FAO impairs stemness and tumorigenesis^28–30^. In GC, the facilitated FAO is supported to aggravate the omental metastasis^31^. Interestingly, a recent study points out that MSC co-culture activates FAO in GC cells, leading to enhanced chemo-resistance^32^. However, mechanism of MSC-regulated FAO in GC remains to be further explored.

Long non-coding RNAs (lncRNAs), a series of RNA transcripts without functional protein products^33,34^, are tightly linked to cancer-related metabolism and chemo-resistance^35,36^. For example, the HOTAIR/miR-17-5p/PTEN axis regulates the chemo-sensitivity in GC^37^. Knockdown of HULC facilitates apoptosis and alleviates chemo-resistance in GC^38^. SNHG16 facilitates colorectal cancer progression through participating in lipid metabolism^39^. MACC1-AS1 enhances glycolysis to contribute to GC progression^40^. Moreover, MACC-AS1 is induced by MSC co-culture and promotes fatty acid oxidation in GC^32^. LncRNA HCP5 has been verified to elicit tumor-promoting function in lung adenocarcinoma^41^, colorectal cancer^42^, and thyroid carcinoma^43^. However, whether HCP5 modulates FAO and chemo-resistance in GC remains elusive.

Current study investigated the relation of HCP5 with GC, demonstrating that HCP5 was induced in GC under MSC-culture and facilitated stemness and chemo-resistance in GC cells. Mechanistically, we demonstrated that HCP5 sponged miR-3619-5p to induce PPARGCA1, leading to the PGC1α/CEBPB-mediated transactivation of CTP1 and facilitating FAO in GC cells.

## Materials and methods

### Cell culture

Human GC cells (AGS and MKN45), human renal epithelial cell (293T) and adult bone marrow MSCs were obtained from American Type Culture Collection (ATCC, Manassas, VA, USA). Cells were maintained with RPMI 1640 medium (Thermo Fisher Scientific, Waltham, MA, USA) adding 10% fetal bovine serum (FBS; Thermo Fisher Scientific) and 1% penicillin-streptomycin (Thermo Fisher Scientific). Cells were cultured under standard conditions of 5% CO_2_ at 37 °C. Transwell cell culture chambers (Millipore, Billerica, MA, USA) were applied for co-culture. In the co-culture system, MSCs were placed on the upper chamber, with GC cells on the lower chamber, allowing direct contact of MSCs with GC cells. 5-fluorouracil (5-FU; CSNpharm, Shanghai, China), oxaliplatin (CSNpharm), calcium folinatc (Xudong, Shanghai, China) and etomoxir (ETX; CSNpharm) were applied to treat cells. CD44, CD133, CD29 and CD90 antibodies were purchased from MiltenyiBiotec (Somerville, MA, USA).

### Sphere-forming assay

Cells were placed in 96-well plates and propagated in the serum-free DMEM/F12 (Invitrogen, Carlsbad, CA, USA) adding 10 μg/mL of heparin (Sigma-Aldrich, St. Louis, MO, USA), 20 ng/mL of epidermal growth factor (EGF; Peprotech, Rocky Hill, NJ, USA), 20 ng/mL of basic fibroblast growth factor (bFGF; Peprotech), 1% B-27 (Gibco, Rockville, MD, USA), and 1x Penicillin-Streptomycin Solution (Sigma-Aldrich). The number of spheres was evaluated after 1 week.

### Cell transfection

The pcDNA3.1 vector targeting HCP5 and the empty vector, specific shRNA against PPARGC1A (sh-PPARGC1A) and its corresponding NC (sh-NC) were acquired from Genechem (Shanghai, China). Besides, miR-3619-5p mimic and NC mimic were obtained from GenePharma (Shanghai, China). Cells were transfected with these plasmids via Lipofectamine 3000 (Invitrogen).

### RT-qPCR

Isolation of total RNA was conducted by TRIzol reagent (Invitrogen) and reverse transcription of RNA to cDNA was implemented by RevertAid First Strand cDNA Synthesis Kit (Thermo-Fisher Scientific). RT-qPCR was carried out on the ABI PRISM 7300 Sequence Detection system (Applied Biosystems, Foster City, CA, USA) by the application of SYBR® Premix Ex Taq™ II (Takara, Dalian, China) with Taqman UniversalMaster Mix II (Life Technologies Corporation, Carlsbad, CA, United States) referring to the manufacturers’ instructions. Calculation of gene expressions relative to GAPDH or U6 was based on 2^−ΔΔCt^ method.

### Western blot

According to previous description, western blot was conducted^44^. Following primary antibodies were acquired from Abcam (Cambridge, USA): SOX2 (ab97959), Oct4 (ab181557), LIN28 (ab46020), CD133 (ab19898), CPT1 (ab189182), ACS (ab177958), p-AMPK (ab23875), AMPK (ab32047), PGC1α (ab54481), GAPDH (ab125247).

### Flow cytometry

For studying the rate of CD44^+^ or CD133^+^ cells, cells were washed twice by phosphate-buffered saline (PBS; Sigma-Aldrich). Upon that, cells were fixed in bovine serum albumin (BSA; Sigma-Aldrich) and stained by use of FITC Mouse anti-CD44 (Abcam) or anti-CD133 (Abcam). A FACS Aria II instrument (BD Biosciences, Franklin Lakes, NJ, USA) was used for Flow Cytometry analysis. For exploring cell apoptosis, Annexin-V-FITC apoptosis detection kit (BD Biosciences) was utilized. Cells were adjusted in binding buffer (Invitrogen). Annexin V and propidium iodide (PI; Invitrogen) were applied to stain cells for 10 min in the dark, and binding buffer was supplemented to cell suspension. Apoptosis cells were finally detected by FACS Calibur (BD Biosciences). For analyzing cell cycle, cells with indicated treatment and transfections were stained with PI, followed by the analysis on flow cytometer (FACScan; BD Biosciences, USA) installed with the Cell Quest software (BD Biosciences).

### Colony forming assay

Cells were placed to six-well plates and cultured for 14 days. Upon fixation with 4% formaldehyde (Sigma-Aldrich), colonies were stained in 1% crystal violet (Sigma-Aldrich). Colonies were photographed and counted.

### Animal assays

BALB/C nude mice (4-week-old) were bought from Charles River Laboratories (Wilmington, MA, USA) for carrying out the following two assays. In the first assay, continuous dilutions of MKN45 cell suspensions (5 × 10^5^, 5 × 10^4^, and 5 × 10^3^ cells) with or without 5 × 10^6^ MSCs were subcutaneously injected into nude mice. Mice were euthanized 6 weeks later, and tumor formation was evaluated. Besides, cells were transfected with pcDNA3.1/HCP5 or pcDNA3.1 and intraperitoneally injected into mice treated with or without FOLFOX (oxaliplatin 6 mg/kg followed 2 h later by 5-FU 50 mg/kg and calcium folinatc 90 mg/kg) weekly. Tumors volume was calculated every 4 days. After 4 weeks, mice were killed and tumors weight was measured. Animal experiments were approved by the ethic committee of the Second Hospital of Shandong University.

### Tissue samples

Total 45 pairs of GC tissues and adjacent normal tissue were acquired from patients at the Second Hospital of Shandong University. Tissue samples were frozen at −80 °C right after surgical excision. Experimental procedures have been approved by the Ethics Committee of the Second Hospital of Shandong University. Written informed consents were attained from patients who had received neither chemotherapy nor radiotherapy before surgery.

### RNA pull-down

RNA pull-down was implemented as previously described^45^.

### RNA immunoprecipitation

RNA immunoprecipitation (RIP) was conducted via the Magna RIP™ RNA-Binding Protein Immunoprecipitation Kit (Millipore). Anti-Argonaut 2 (Ago2) antibody (Millipore) or anti-IgG antibody (Millipore) was applied. The co-precipitated RNAs were tested with RT-qPCR.

### Luciferase reporter assay

HCP5-WT/Mut or PPARGC1A-WT/Mut was sub-cloned into the pmirGLO dual-luciferase plasmid (Promega, Madison, WI, USA) to construct pmirGLO-HCP5-WT/Mut or pmirGLO-PPARGC1A-WT/Mut which was then co-transfected into 293 T cells with miR-3619-5p mimic or NC mimic. CPT1 promoter was sub-cloned into the pGL3-basic vector (Promega) to generate pGL3-CPT1 promoter which was subsequently co-transfected into 293T or MKN45 cells with indicated transfection plasmids. Luciferase activities were examined via Dual-Luciferase Reporter Assay System (Promega).

### CPT1 activity assay

CPT1 activity assay was carried out based on previous description^46^.

### Fatty acid β-oxidation rate detection

Detection of Fatty Acid (FA) β-oxidation rate was conducted in line with previous description^47^.

### Detection of ATP levels

ATP levels were assessed via the Firefly Luciferase ATP Assay Kit (Beyotime, Shanghai, China). Luminance was determined by use of a SpectraMax M5 microplate reader (Molecular Devices, Sunnyvale, CA, USA).

### Cell counting Kit-8 (CCK-8) assay

Cells inoculated to 96-well plates were treated with various concentrations of oxaliplatin or 5-FU for 48 h. Then, 10 μL Cell counting Kit-8 (CCK-8) reagent (Beyotime) was added at indicated time points. Absorbance at 450 nm was finally examined.

### Chromatin immunoprecipitation (ChIP)

Chromatin immunoprecipitation (ChIP) was conducted by an EZ ChIP Chromatin Immunoprecipitation Kit (Millipore). Briefly, PGC1α antibody was adopted for immunoprecipitation of chromatin, and IgG was negative control. Isolated RNA was assayed by RT-qPCR.

### Co-immunoprecipitation

Co-immunoprecipitation (Co-IP) was implemented in line with previous description^48^.

### Immunohistochemistry

Immunohistochemistry (IHC) was carried out with the use of primary antibodies against CD29 (Abcam), CD90 (Abcam), PGC1α, CTP1, and Ki-67 (Abcam) following prior description^49^.

### Hematoxylin-Eosin staining

To calculate metastatic nodules, tumors in mice were collected and washed with PBS, followed by fixed in 10% neutral formalin solution (Sigma-Aldrich) and embedded in paraffin (Sigma-Aldrich). Tissues were sliced, after which were stained by Hematoxylin-Eosin (HE) (Sigma-Aldrich).

### Statistical analysis

Data determined as mean ± standard deviation (S.D.) were assayed via SPSS 23.0 (IBM, Armonk, NY, USA). Experiments were conducted for thrice. One-way/two-way analysis of variance (ANOVA) or Student’s *t*-test was used for difference analysis. *P* < 0.05 indicated that data was statistically significant.

## Results

### MSC improved stem properties and chemo-resistance in GC cells

**Fig. 1:**
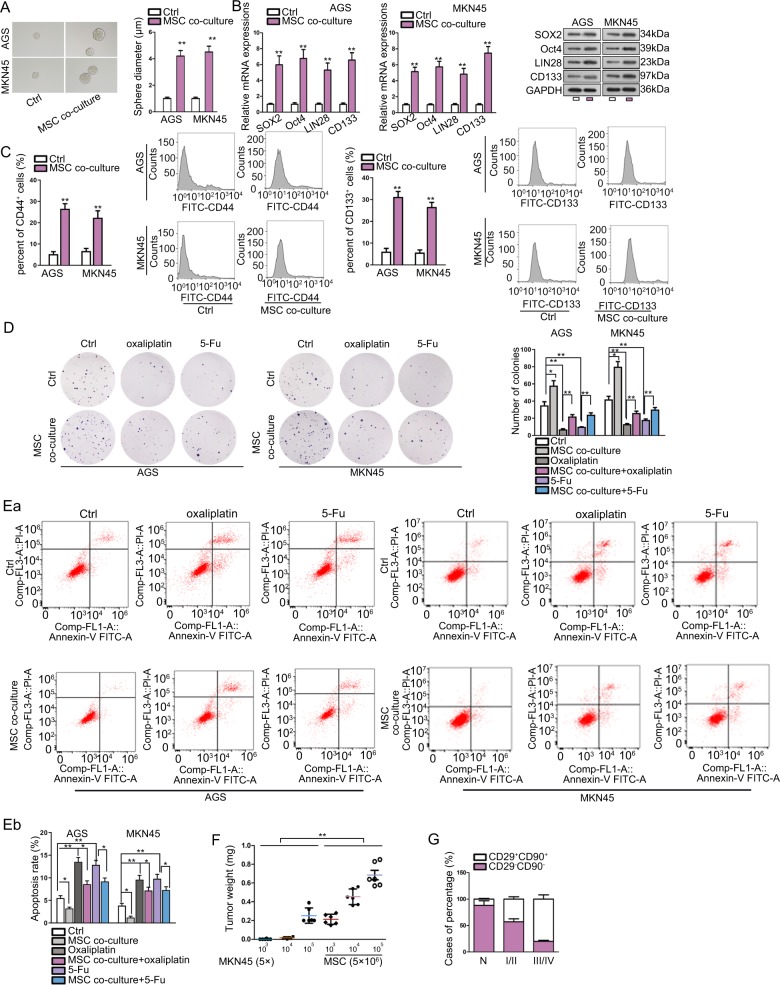
MSC improved stem properties and chemo-resistance in GC cells. **a** Representative images and quantification of the tumor spheres generated by AGS and MKN45 cells with or without the co-culture with MSCs. **b** RT-qPCR and western blot results of stemness markers including SOX2, OCT4, LIN28 and CD133 in AGS and MKN45 cells co-cultured with or without MSCs. **c** Pictures and quantification of flow cytometry analysis of the rate of CD133^+^ and CD44^+^ GC cells cultured with or without MSCs. **d** GC cells co-cultured with or without MSCs were treated with oxaliplatin or 5-Fu. Pictures of colonies generated by GC cells of each group. **e** Flow cytometry analysis of apoptotic GC cell of each group. **f** Nude mice were injected with MKN45 cells at the count of 10^3^, 10^4^, 10^5^ cells, with or without the mixture with MSCs. Tumor weight in mice of each group after 28 days were assessed. **g** Quantification of IHC staining rate of CD29^+^CD90^+^ in non-tumorous specimens or GC specimens from patients at stage I/II and stage III/IV. *P < 0.05, **P < 0.01. Error bars indicate SD. Each assay was conducted for three times.

First, we co-cultured two GC cell lines (MNK45 and AGS) with MSCs to examine the impact of MSCs on GC cell stemness and chemo-resistance. Consequently, the ability of GC cells to generate tumor spheres was enhanced under the co-culture with MSC (Fig. 1a). In addition, stemness markers (SOX2, OCT4, LIN28, and CD133) were examined by RT-qPCR and western blot. Data presented that MSCs co-culture induced the level of these markers in GC cells (Fig. 1b). Flow cytometry analysis showed that rate of CD133^+^ and CD44^+^ GC cells increased under the co-culture with MSCs (Fig. 1c). Cellular response to drugs in GC was determined by colony formation and flow cytometry apoptosis analysis. We observed that MSCs co-culture induced colony formation ability of GC cells, and counteracted the repressing effect of oxaliplatin and 5-Fu on the colony formation of GC cells (Fig. 1d). Apoptosis of GC cells was suppressed by MSCs co-culture, and the inductive effect of oxaliplatin and 5-Fu on GC cell apoptosis was abrogated by MSCs co-culture (Fig. 1e). As presented by Fig. S1A, cell cycle analysis revealed that MSC co-culture reduced the proportion of cells at G0/G1 phase, and induced the rate of cells at S and G2/M phases, indicating that cell cycle was accelerated in GC cells co-cultured with MSCs. Oxaliplatin or 5-Fu treatment caused cell cycle arrest at G0/G1 phase, and MSC co-culture alleviated such effect in GC cells. These findings indicated that MSCs conferred chemo-resistance of GC cells. Next, we tried to consolidate the results through animal models. MKN45 cells were injected into nude mice with the count of 5 × 10^3^, 5 × 10^4^, or 5 × 10^5^, mixed with or without 5 × 10^6^ MSCs. Consequently, we observed that injection of MKN45 cells alone with the count of 5 × 10^3^, 5 × 10^4^ cells hardly generated tumors in mice, whereas the mixture of MSCs increased the weight of tumors grafted by MKN45 cells in mice (Fig. 1f). Besides, we confirmed through IHC staining that GC specimens (*n* = 45) presented higher stain positivity of CD29 and CD90 compared to non-tumorous tissues, and the rate of CD29^+^CD90^+^ in GC specimens was positively correlated with clinical stage (Fig. 1g). Collectively, it was suggested that MSCs improved stem properties and chemo-resistance in GC cells.

### **MSC-induced** lncRNA HCP5 facilitated stemness and chemo-resistance in GC cells

**Fig. 2:**
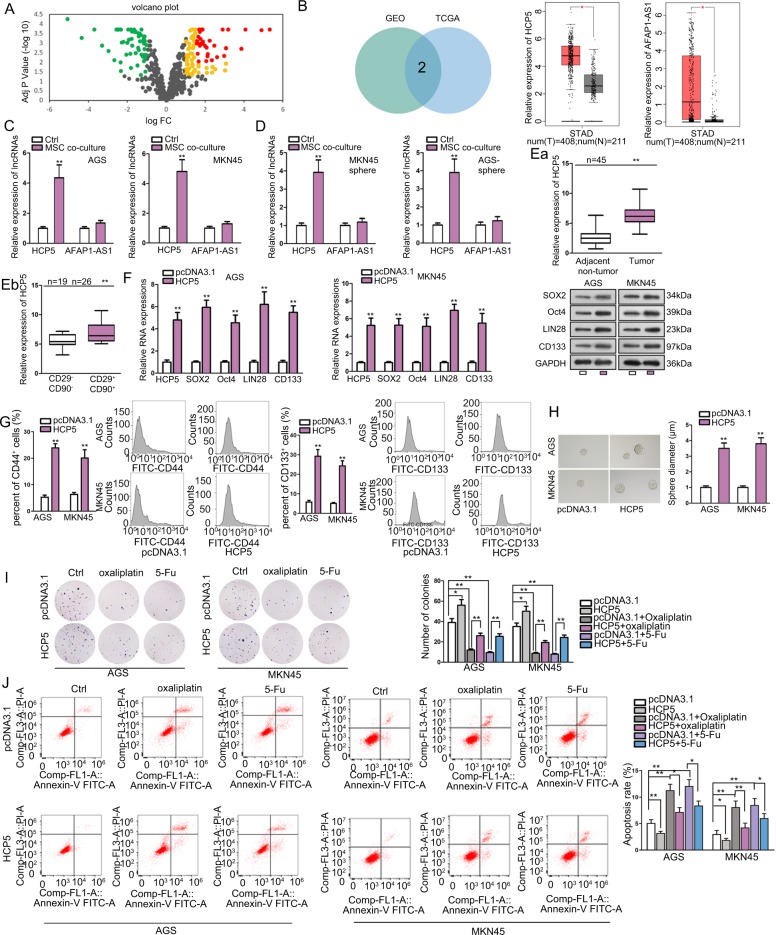
MSC-induced lncRNA HCP5 facilitated stemness and chemo-resistance in GC cells. **a** Volcano plot showed the levels of metabolic pathway-related lncRNAs in GC samples versus para-cancerous tissues according to GEO data (GEO accession: GSE96856). Yellow+red: 84 lncRNAs were upregulated in GC samples versus para-cancerous tissues (*P* < 0.05, Log FC > 1). Green: 56 downregulated lncRNAs in GC samples (*P* < 0.05, Log FC < −1). Red: 31 most significantly upregulated lncRNAs in GC samples (P < 0.01, Log FC > 1.5). **b** Among abovementioned 31 lncRNAs, only HCP5 and AFAP1-AS1 were upregulated in stomach adenocarcinoma (STAD) samples in GEPIA database. **c**, **d** RT-qPCR results of HCP5 and AFAP1-AS1 levels in GC cells and generated tumor spheres under the co-culture with MSCs versus control. **e** RT-qPCR analysis of HCP5 level in GC tissues (*n* = 45) versus matched para-tumorous tissues (*n* = 45), and in CD29^+^CD90^+^ GC tissues (*n* = 26) versus CD29^−^CD90^−^ GC tissues (*n* = 19). **f**. RT-qPCR and western blot data of HCP5 and stemness markers in GC cells under HCP5 overexpression versus control. **g**, **h** Flow cytometry analysis of CD133^+^ and CD44^+^ rate in GC cells and sphere formation of GC cells under HCP5 overexpression. **i**, **j** AGS and MKN45 cells were transfected with pcDNA3.1 or pcDNA3.1/HCP5 and treated with oxaliplatin or 5-Fu. Colony formation and flow cytometry apoptosis analysis were applied to evaluate the chemo-resistance of GC cells in each group. *P < 0.05, **P < 0.01. Error bars indicate SD. Each assay was conducted for three times.

Later, we explored the mechanism underlying the regulation of MSCs on GC cell stemness and chemo-resistance. Chemo-resistance is often developed along with the dysregulation of gene expression and alteration of metabolisms. LncRNAs are recognized to be deeply implicated in the regulation of chemo-resistance and metabolism of cancer cells^35,36^. Therefore, we deduced that MSCs could regulate certain lncRNAs related to metabolism in GC cells. Therefore, we analyzed the expression of metabolic pathways-related lncRNAs in GC based on GEO data (GEO accession: GSE96856). As a result, we found that 84 lncRNAs were upregulated in GC samples versus para-cancerous tissues (*P* < 0.05, Log FC > 1), and 56 were downregulated (*P* < 0.05, Log FC < −1) (Fig. 2a). Then, we picked 31 most significantly upregulated lncRNAs in GC (*P* < 0.01, Log FC > 1.5) and analyzed their expressions in TCGA database through GEPIA tool (http://gepia.cancer-pku.cn/). Results showed that only HCP5 and AFAP1-AS1 were upregulated in stomach adenocarcinoma (STAD) samples compared with normal samples (Fig. 2b). To further probe the association of these two lncRNAs with the impact of MSCs on GC cells, we detected their expressions in GC cells under MSCs co-culture. It turned out that in both GC cells and the tumor spheres generated by GC cells, the expression of HCP5, rather than AFAP1-AS1, was upregulated under the co-culture with MSCs versus control (Fig. 2c, d). In addition, we found that HCP5 was upregulated in GC tissues versus (*n* = 45) the matched para-tumorous tissues (*n* = 45) (Fig. 2e). By analyzing the expression of CD29 and CD90, well-known MSC surface antigen markers, we found that HCP5 was highly expressed in CD29^+^CD90^+^ GC tissues (*n* = 26) compared with the CD29^−^CD90^−^ GC tissues (*n* = 19) (Fig. 2e). Thus, we speculated that HCP5 might participate in the influence of MSCs on GC cell stemness and chemo-resistance.

Then, the impact of HCP5 on the stem cell properties and drug resistance of GC cells was detected. RT-qPCR and western blot analyses confirmed that HCP5 and the stemness genes were overtly upregulated in GC cells upon the transfection of pcDNA3.1/HCP5 (Fig. 2f). Rate of CD44^+^ and CD133^+^ GC cells increased upon the overexpression of HCP5 (Fig. 2g). Besides, upregulating HCP5 in GC cells induced sphere formation (Fig. 2h). Further, forced expression of HCP5 enhanced colony formation and abrogated the suppressive effect of oxaliplatin and 5-Fu on colony formation in GC cells (Fig. 2i). Apoptosis of GC cells was inhibited by HCP5 overexpression, and the apoptosis induced by oxaliplatin and 5-Fu in GC cells was reversed by the ectopic expression of HCP5 (Fig. 2j). According to cell cycle analysis in Fig. S2A, HCP5 overexpression accelerated cell cycle, but oxaliplatin and 5-Fu arrested GC cells at G0/G1 phase. Overexpressing HCP5 reversed the cell cycle arrest at G0/G1 phase caused by oxaliplatin and 5-Fu in GC cells. Jointly, it was indicated that MSC-induced lncRNA HCP5 facilitated stemness and chemo-resistance in GC cells.

### HCP5 interacted with miR-3619-5p to regulate stemness and chemo-resistance in GC

**Fig. 3:**
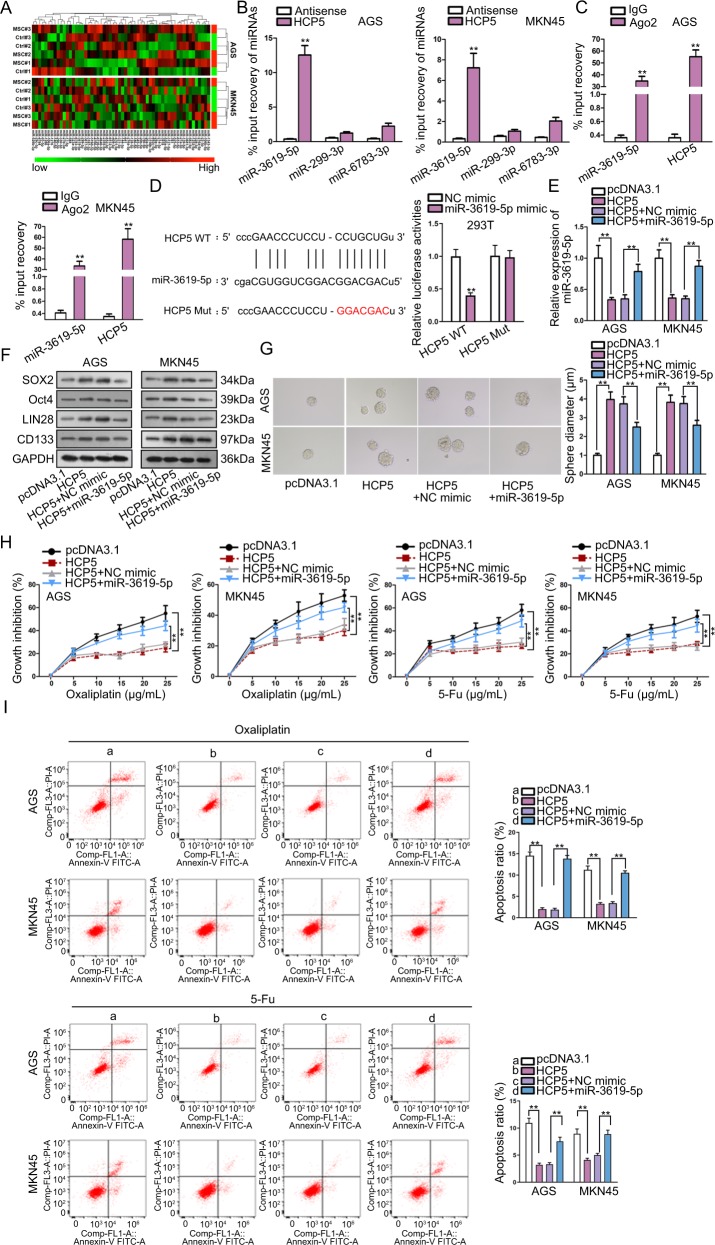
HCP5 interacted with miR-3619-5p to regulate stemness and chemo-resistance in GC. **a** RT-qPCR analysis revealed that 3 most downregulated miRNAs in GC cells responding to co-culture with MSCs were miR-3619-5p, miR-299-3p, and miR-6873-3p. **b** Pull down assay depicted that only miR-3619-5p could be pulled down by HCP5. **c** RIP analysis demonstrated the co-immunoprecipitation of HCP5 and miR-3619-5p. **d** MiR-3619-5p sites on HCP5 were predicted by starBase3.0, the mutated sites were designed for luciferase reporter assay. Luciferase activity of HCP5 WT/Mut was assessed in 293T cells under overexpression of miR-3619-5p versus control. **e**, **f** AGS and MKN45 cells were transfected with pcDNA3.1, pcDNA3.1/HCP5 (named HCP5), HCP5 + NC mimic, or HCP5 + miR-3619-5p mimic. RT-qPCR results of miR-3619-5p and western blot results of stemness markers in GC cells of each group. **g** Pictures of tumor spheres and quantification in GC cells of each group. **h** GC cells with indicated transfection were treated with oxaliplatin or 5-Fu at indicated doses. CCK-8 was applied to assess chemo-resistance of GC cells of each group. **i** Flow cytometry analysis of GC cell apoptosis of each group. **P < 0.01. Error bars indicate SD. Each assay was conducted for three times.

Next, regulatory mechanism of HCP5 in GC was explored. As widely reported, lncRNAs can function as miRNA sponge to regulate chemo-resistance in cancer cells^36,37^. Therefore, we searched for the target miRNAs for HCP5. Prediction results from starBase3.0 (http://starbase.sysu.edu.cn/) showed that HCP5 potentially interacted with 58 miRNAs. RT-qPCR analysis revealed that the three most significantly downregulated miRNAs in GC cells responding to the co-culture with MSCs were miR-3619-5p, miR-299-3p, and miR-6783-3p (Fig. 3a), indicating their involvement in the regulation of MSC on GC cells. Pull down assay depicted that only miR-3619-5p could be pulled down by HCP5 instead of antisense HCP5 in GC cells (Fig. 3b), indicating that HCP5 interacted with miR-3619-5p. Therefore, we deduced that miR-3619-5p was a target for HCP5 in GC. RIP analysis demonstrated the co-immunoprecipitation of HCP5 and miR-3619-5p in GC cells (Fig. 3c). The interacting sequences on HCP5 for miR-3619-5p and the mutated sequences were presented in Fig. 3d. Luciferase activity of HCP5 WT was reduced by miR-3619-5p mimic, whereas that of HCP5 Mut exhibited no significant variation (Fig. 3d). Moreover, we tried to examine whether HCP5 modulated stemness and chemo-resistance in GC cells through miR-3619-5p. RT-qPCR data confirmed that overexpression of HCP5 reduced miR-3619-5p expression, whereas co-transfection of miR-3619-5p mimic recovered miR-3619-5p level in GC cells (Fig. 3e). The protein levels of stemness markers induced by HCP5 overexpression were repressed by the co-transfection of miR-3619-5p mimic in GC cells (Fig. 3f). Ability of GC cells to form tumor spheres was enhanced by HCP5 overexpression and repressed by miR-3619-5p overexpression (Fig. 3g). CCK-8 assay demonstrated that the inhibitive effect of oxaliplatin and 5-Fu on GC cell viability was reduced by HCP5 overexpression and regained by the forced expression of miR-3619-5p (Fig. 3h). Also, under the treatment of oxaliplatin and 5-Fu, apoptosis in GC cells was reduced by HCP5 overexpression, and rescued by miR-3619-5p overexpression (Fig. 3i). In oxaliplatin or 5-Fu treated GC cells, overexpressing HCP5 reduced cell rate at G0/G1 phase and increased cell rate at S and G2/M phases, whereas overexpressing miR-3619-5p reversed such effect (Fig. S3A), indicating that miR-3619-5p overexpression repressed the accelerating effect of HCP5 overexpression on GC cell cycle. Hence, it was suggested that HCP5 interacted with miR-3619-5p to regulate stemness and chemo-resistance in GC.

### MSC-activated HCP5/miR-3619-5p axis regulated FAO in GC

**Fig. 4:**
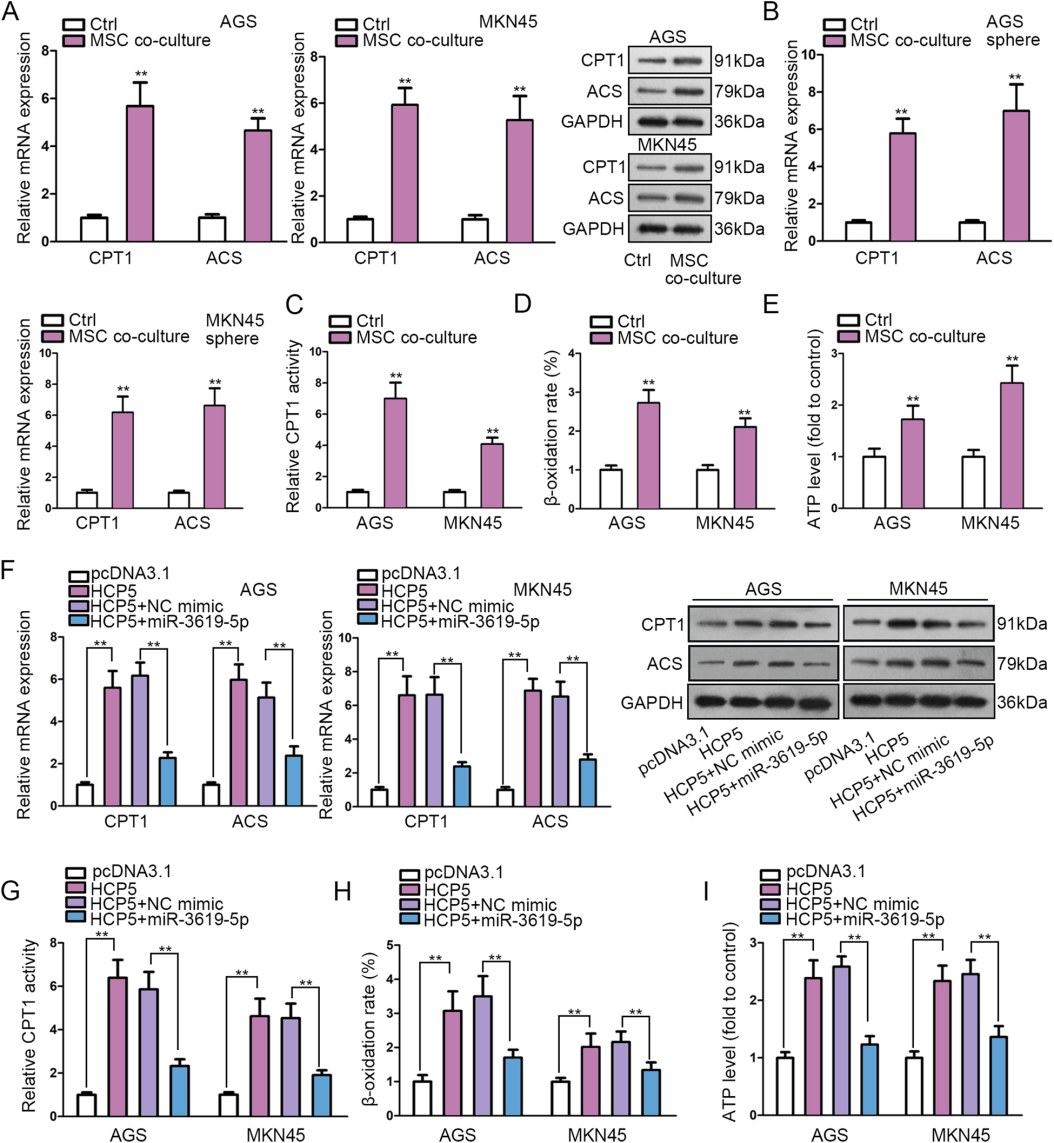
MSC-activated HCP5/miR-3619-5p axis regulated FAO in GC. **a**, **b** MSC co-culture induced the FAO-associated enzymes (CPT1 and ACS) in GC cells and GC cell-derived tumor spheres. **c–e** The activity of CPT1, β-oxidation rate, and ATP level in GC cells were facilitated as a result of MSC co-culture. **f** RT-qPCR and western blot data of the levels of CPT1 and ACS in AGS and MKN45 cells under indicated transfection. **g–i** CPT1 activity, β-oxidation rate and ATP level of GC cells with indicated transfection. **P < 0.01. Error bars indicate SD. Each assay was conducted for three times.

Later, we detected the downstream mechanism of HCP5/miR-3619-5p in GC. MiRNAs are axiomatically recognized as gene modulators considering that they functioned through base-pair binding with target mRNAs^50^. Therefore, to annotate the function of miR-3619-5p, we obtained the target genes for miR-3619-5p in starBase3.0 and carried out KEGG pathway analyzed these genes through online bioinformatics tool DAVID6.8 (https://david.ncifcrf.gov/). Results presented that miR-3619-5p target genes were significantly enriched in FAO metabolism (Supplementary Table 1). FAO has been uncovered to play a role in chemo-resistance, stemness, and tumorigenesis in cancer cells^27–30^. Also, it has been proved that the facilitated FAO can induce metastasis, confer chemo-resistance and improve stemness of GC cells^31,32^. Thus, we hypothesized that MSC-activated HCP5/miR-3619-5p regulated FAO in GC cells. We confirmed that MSC co-culture induced the representative FAO-associated enzymes, CPT1 and acetyl-coenzyme A synthetase (ACS) in both GC cells and GC cell-derived tumor spheres (Fig. 4a, b). Moreover, we verified that the activity of CPT1, β-oxidation rate, and ATP level in GC cells were facilitated as a result of MSC co-culture (Fig. 4c–e). These results suggested that MSC facilitated FAO in GC cells. Later, we detected whether HCP5/miR-3619-5p regulated FAO in GC cells. Expectedly, levels of CPT1 and ACS were induced by HCP5 upregulation and such induction was abrogated by miR-3619-5p overexpression in GC cells (Fig. 4f). Also, promoting effect of HCP5 overexpression on CPT1 activity, β-oxidation rate, and ATP level in GC cells was impaired by the ectopic expression of miR-3619-5p (Fig. 4g–i). In sum, data above revealed that MSC-activated HCP5/miR-3619-5p axis regulated FAO in GC.

### HCP5/miR-3619-5p induced PPARGC1A and PGC1α interacted with CEBPB to induce CPT1 transcription

**Fig. 5:**
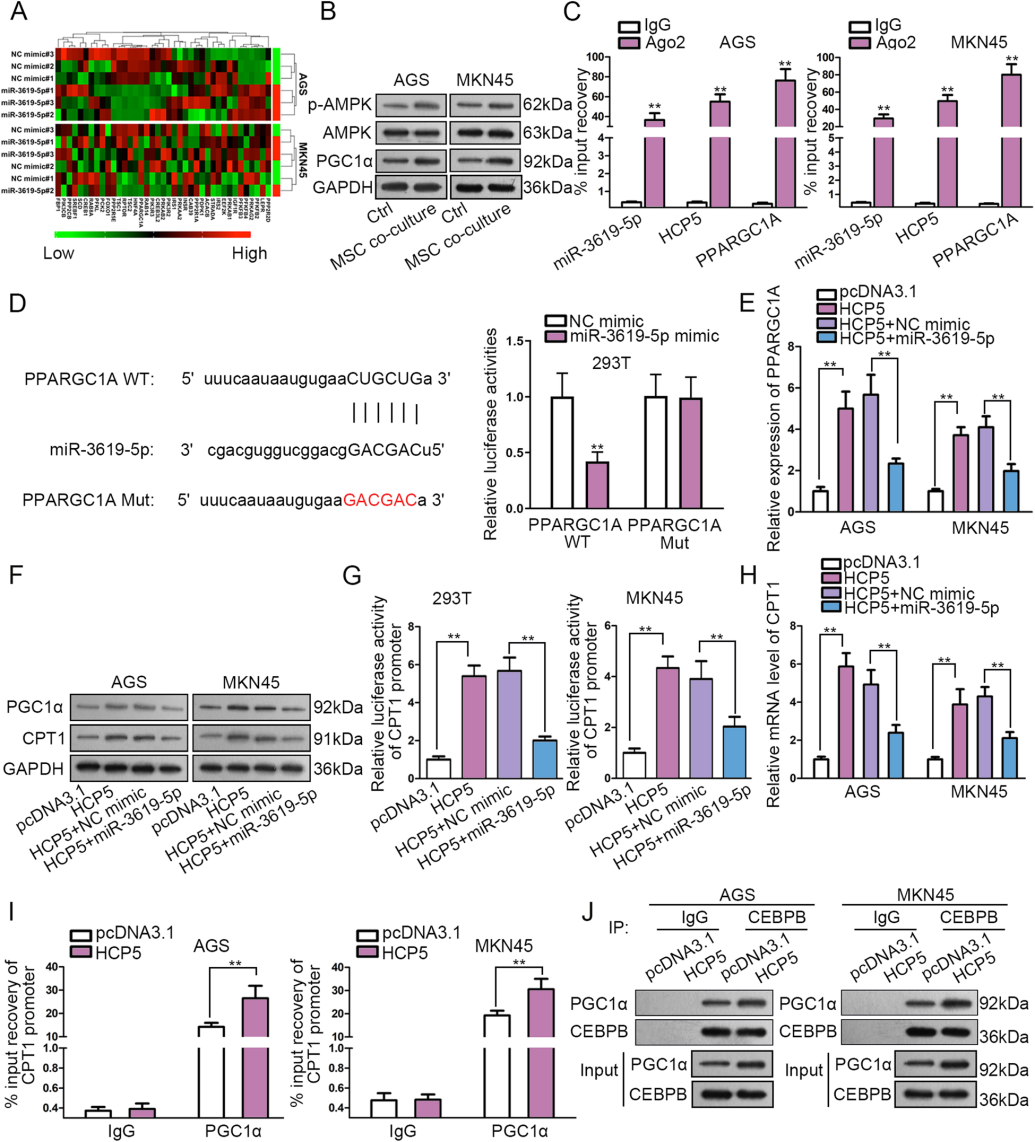
HCP5/miR-3619-5p induced PPARGC1A and PGC1α interacted with CEBPB to induce CPT1 transcription. **a** According to KEGG pathway analysis by DAVID6.8, 39 miR-3619-5p target genes related to AMPK pathway were sort out. RT-qPCR results of 39 gene levels under miR-3619-5p overexpression versus control in GC cells. **b** Western blot results of the levels of p-AMPK and PGC1α in GC cells with or without MSC co-culture. **c** MiR-3619-5p, PPARGC1A mRNA, and HCP5 were all enriched in the immunoprecipitated products of Ago2. **d** MiR-3619-5p sites on PPARGC1A were predicted by starBase3.0, the mutated sites were designed for luciferase reporter assay. Luciferase activity of PPARGC1A WT/Mut was assessed in 293T cells under overexpression of miR-3619-5p versus control. **e**, **f** RT-qPCR results of PPARCG1A level and western blot results of PGC1α and CPT1 level in GC cells with indicated transfection. **g** Luciferase activity of CPT1 promoter reporter in 293T cells under indicated transfection. **h** RT-qPCR data showed the levels of PPARGC1A mRNA in GC cells of each group. **i** ChIP assay revealed that overexpression of HCP5 led to increased enrichment of CPT1 promoter in PGC1α precipitates. **j** CoIP analysis demonstrated that PGC1α protein in CEBPB precipitates was enhanced by enhancing HCP5, with the expression of CEBPB unchanged. **P < 0.01. Error bars indicate SD. Each assay was conducted for three times.

Furthermore, we asked how HCP5/miR-3619-5p regulated FAO in GC. According to results of KEGG analysis, we also found that miR-3619-5p was significantly related to AMPK pathway (Supplementary Table 1). Multiple studies have delineated that AMPK is a key signaling involved in various cancer metabolisms including FAO^51–54^. Combining our previous discovery that miR-3619-5p was related to FAO pathway and that HCP5 facilitated FAO in GC cells through miR-3619-5p, we deduced that miR-3619-5p regulated AMPK pathway by targeting certain genes involved in AMPK signaling. We sorted out 39 miR-3619-5p target genes related to AMPK pathway based on KEGG analysis, and detected their expressions under miR-3619-5p overexpression. We found that the most significantly downregulated gene responding to miR-3619-5p overexpression in GC cells was PPARGC1A (Fig. 5a). PPARGC1A is an important regulator in AMPK pathway, and its protein product, PGC1α is an transcriptional co‐activator responsible for the lipid metabolism, FAO^55^. Additionally, we confirmed that MSC co-culture activated AMPK pathway by observing that the levels of p-AMPK and PGC1α were induced by MSC co-culture (Fig. 5b). Thus, we postulated that HCP5/miR-3619-5p regulated PPARGC1A to affect FAO in GC. RIP assay revealed that miR-3619-5p, PPARGC1A mRNA, and HCP5 were all enriched in the immunoprecipitated products of Ago2 (Fig. 5c). The miR-3619-5p sites on PPARGC1A were predicted through starBase3.0, and were substituted with complementary sequences to generate PPARGC1A Mut reporter for luciferase reporter assay (Fig. 5d). Results displayed that overexpressing miR-3619-5p led to the diminishment of luciferase activity on PPARGC1A WT rather than PPARGC1A Mut (Fig. 5d).

Furthermore, it has been reported that PGC1α can form a transcription complex with CEBPB to activate CPT1 transcriptionally and facilitate FAO in nasopharyngeal carcinoma^56^. We tried to detect whether HCP5/miR-3619-5p axis regulated FAO through this manner. First, we confirmed that PPARCG1A level increased upon HCP5 overexpression and such impact was offset by miR-3619-5p overexpression in GC cells (Fig. 5e). Western blot analysis validated that PGC1α and CPT1 levels were induced by forced HCP5 expression and such effect was counteracted by miR-3619-5p mimic (Fig. 5f). Furthermore, we found that overexpressing HCP5 induced the activity of CPT1 promoter reporter, and miR-3619-5p overexpression reversed such effect (Fig. 5g). Besides, the mRNA level of CPT1 induced by HCP5 overexpression was repressed by miR-3619-5p in GC cells (Fig. 5h). Then, we analyzed whether HCP5 influenced the binding of PGC1α to CPT1 promoter. ChIP assay revealed that overexpression of HCP5 increased the enrichment of CPT1 promoter in PGC1α precipitates (Fig. 5i). Finally, effect of HCP5 on the interaction between PGC1α and CEBPB was tested. CoIP analysis demonstrated that abundance of PGC1α protein in precipitates of CEBPB was enhanced by HCP5 overexpression, with CEBPB expression unchanged (Fig. 5j). Together, these results suggested that HCP5 induced the expression of PGC1α through miR-3619-5p so as to facilitate the transactivation CPT1 by PGC1α/CEBPB complex in GC cells.

### HCP5 regulated PPARGC1A-mediated FAO to facilitate stemness and chemo-resistance in GC cells

**Fig. 6:**
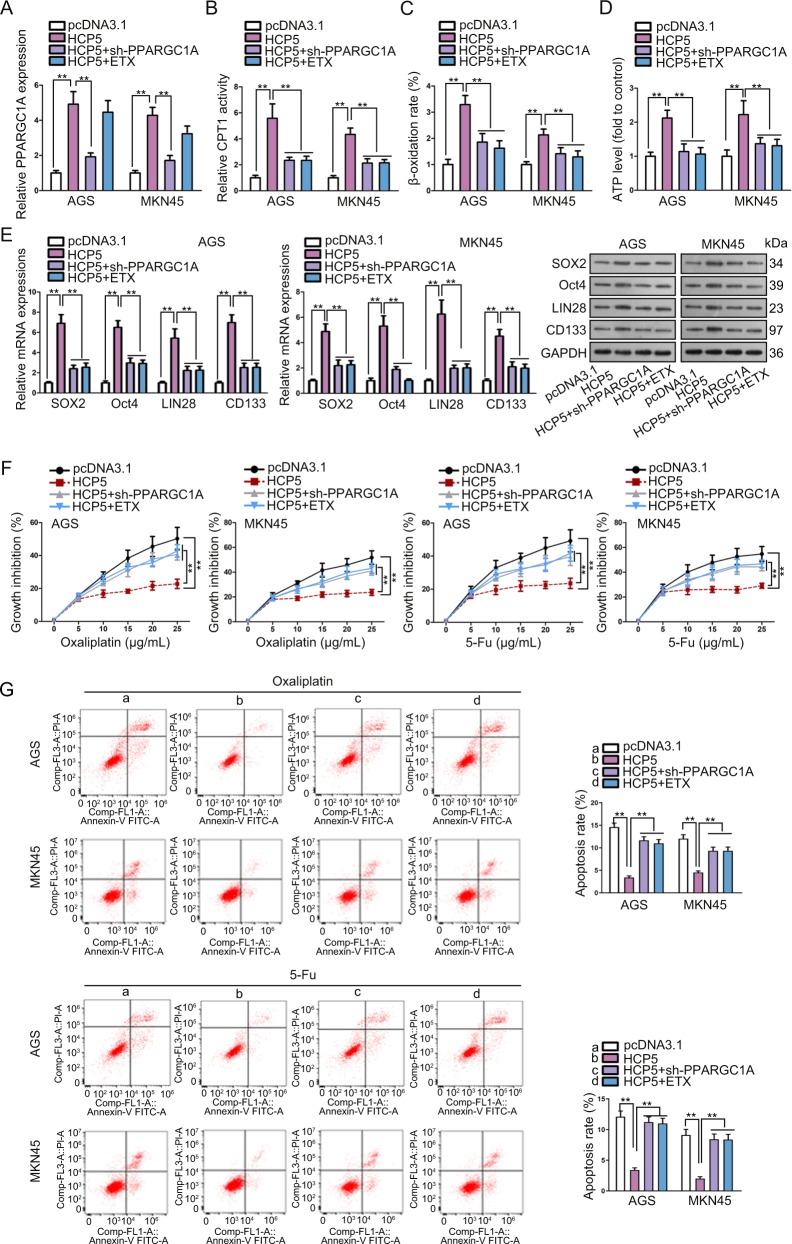
HCP5 regulated PPARGC1A-mediated FAO to facilitate stemness and chemo-resistance in GC cells. AGS and MKN45 cells were transfected with pcDNA3.1, pcDNA3.1/HCP5, pcDNA3.1/HCP5 + sh-PPARGC1A, or pcDNA3.1/HCP5 + ETX treatment. Etomoxir (ETX) was the FAO inhibitor. **a** RT-qPCR results of PPARGC1A expression in GC cells of each group. **b–d** CPT1 activity, β-oxidation rate, and ATP level in GC cells of each group. **e** RT-qPCR and western blot data of stemness markers in GC cells of each group. **f** CCK-8 assay was used to assess the chemo-resistance of GC cell of each group with the treatment of oxaliplatin or 5-Fu at indicated doses. **g** Flow cytometry analysis of apoptosis of GC cells of each group with the treatment of oxaliplatin or 5-Fu. **P < 0.01. Error bars indicate SD. Each assay was conducted for three times.

Rescue experiments were conducted to determine whether HCP5 conferred stemness and chemo-resistance in GC cells through PPARGC1A and FAO. We validated that PPARGC1A expression induced by HCP5 overexpression was decreased by the transfection of sh-PPARGC1A (Fig. 6a). The CPT1 activity, β-oxidation rate, and ATP level induced by HCP5 were abolished by PPARGC1A silence or the addition of etomoxir (ETX), the FAO inhibitor, in GC cells (Fig. 6b–d). The inductive effect of HCP5 overexpression on stemness markers in GC cells was counteracted by sh-PPARGC1A or ETX (Fig. 6e). Moreover, the inhibitory effect of oxaliplatin and 5-Fu on GC cell viability was attenuated by HCP5, and such attenuation was reversed by PPARGC1A silence or ETX addition (Fig. 6f). Under the treatment of oxaliplatin or 5-Fu, the apoptosis of GC cells reduced by HCP5 overexpression was recovered by the PPARGC1A knockdown or ETX treatment (Fig. 6g). Knocking down PPARGC1A or adding ETX reversed the accelerating effect of HCP5 overexpression on GC cell cycle under oxaliplatin or 5-Fu (Fig. S4A). In a word, HCP5 regulated PPARGC1A-mediated FAO to facilitate stemness and chemo-resistance in GC cells.

### HCP5 conferred chemo-resistance of GC in vivo

**Fig. 7:**
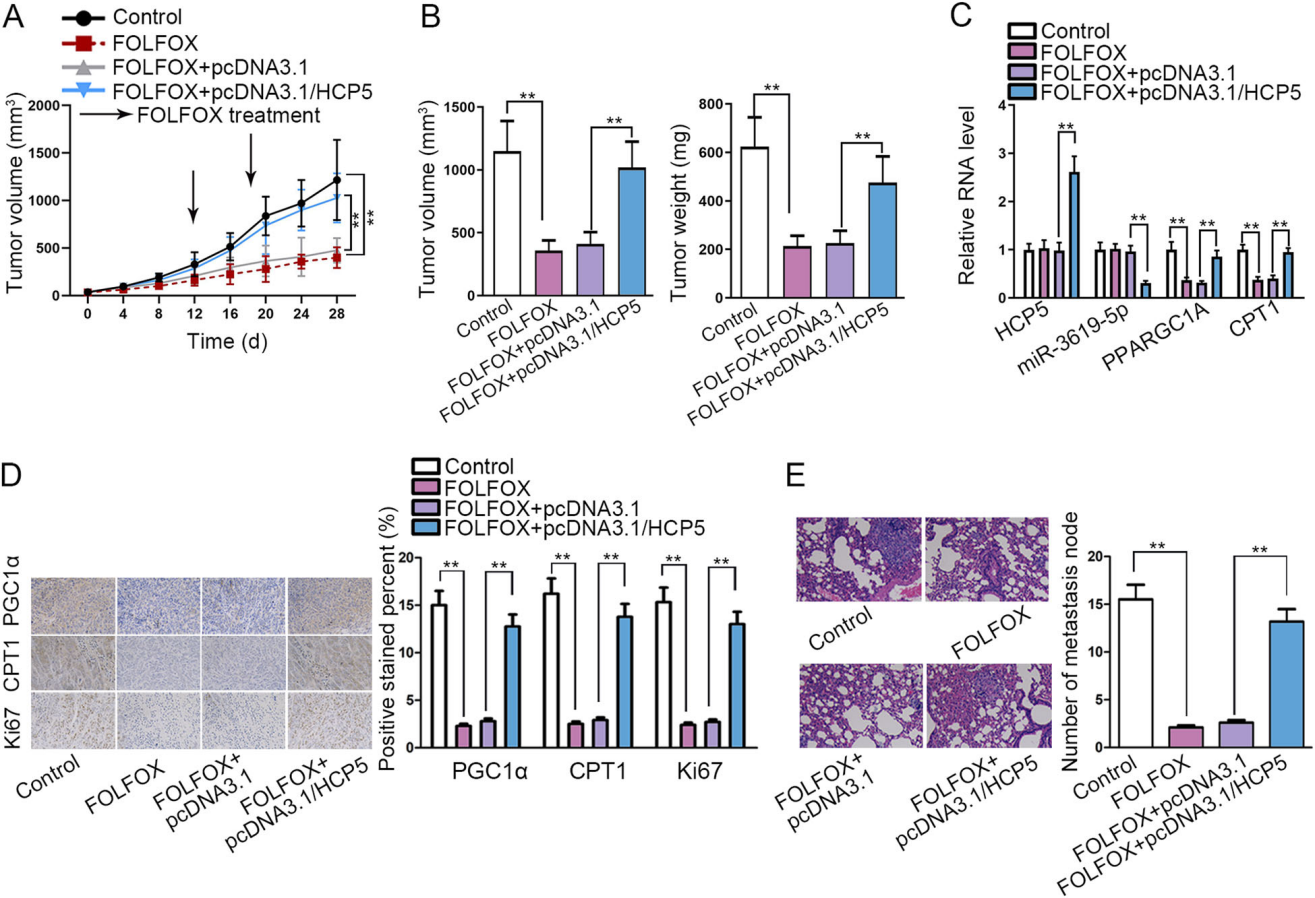
HCP5 conferred chemo-resistance of GC in vivo. MKN45 cells were transfected with pcDNA3.1 or pcDNA3.1/HCP5. Mice were injected with MKN45 cells and received FOLFOX regime weekly (oxaliplatin 6 mg/kg followed 2 h later by 5-florouracil 50 mg/kg and calcium folinatc 90 mg/kg, intraperitoneally (i.p.). **a** Tumor volumes were calculated every 4 days, and the growth curve was drawn. **b** Tumor weight and volume of each group was assessed 28 days after injection. **c** The relative levels of HCP5, miR-3619-5p, PPARGC1A and CPT1 in xenografts of each group were detected by RT-qPCR. **d** IHC staining of PGC1α, CPT1, and proliferation index Ki67 in xenografts of each group. **e** HE staining of the metastatic nodes in lungs of mice of each group. **P < 0.01. Error bars indicate SD. Each assay was conducted for three times.

Finally, we established animal models to detect the effect of HCP5 on chemo-resistance of GC in vivo. MKN45 cells transfected with pcDNA3.1 or pcDNA3.1/HCP5 were mixed with MSCs and injected into nude mice to generate tumors. The mice were separated into two groups: one received FOLFOX treatment 7 days a time, whereas another did not. Results showed that the FOLFOX treatment inhibited GC tumorigenesis in mice, and HCP5 overexpression abrogated the anti-tumor effect of FOLFOX regiment (Fig. 7a). Concordantly, the final tumor weight and volume reduced by FOLFOX in mice were reversed by the overexpression of HCP5 (Fig. 7b). In addition, we found that tumors generated by HCP5-overexpressing MKN45 cells in mice presented higher HCP5 expression and lower miR-3619-5p expression (Fig. 7c). Also, the expressions of PPARGC1A and CPT1 reduced by FOLFOX in xenografts could be rescued by HCP5 overexpression (Fig. 7c). IHC staining displayed a reduced stain positivity of PGC1α, CPT1, and proliferation index Ki67 in xenografts of FOLFOX-treated mice, and such reduction could be reversed by HCP5 overexpression (Fig. 7d). According to HE staining, metastatic nodes reduced by FOLFOX treatment in mice was recovered by the overexpression of HCP5 (Fig. 7e). In conclusion, HCP5 conferred chemo-resistance of GC in vivo.

## Discussion

Resistance of cancer cells to various chemotherapeutic drugs involves diverse mechanisms, such as epigenetic and genetic dysregulation, signaling pathway alterations, as well as cell metabolism^33,57,58^. MSCs are increasingly proved to be tightly linked to the stem cell properties and drug resistance in cancer cells^13–15^, including in GC^18,19^. Therefore, exploration of the mechanism behind the regulation of MSC on GC chemo-resistance may provide new thoughts to develop more efficient treatment regimes in GC. In line with these findings, our study confirmed that MSC co-culture helped GC cells to acquire stem cell properties and confer the resistance to oxaliplatin and 5-Fu.

Former studies have validated the pivotal role of metabolic modulation in cancer progression as well as drug resistance in cancers^59–62^. LncRNAs are demonstrated to pose significant effects on the cancer-related metabolism, such as lipid metabolism and glycolysis, in a variety of cancer types^39,40^. Also, accumulating studies have attributed the development of chemo-resistance to aberrant expression of certain lncRNAs^37,38^. These discoveries suggested that MSCs might regulate chemo-resistance in GC through altering expressions of lncRNAs, specifically the lncRNAs related to metabolic pathways. Herein, we identified the metabolic pathway-related lncRNAs upregulated in GC samples through GEO database and analyzed their expressions in GEPIA, finding that HCP5 and AFAP1-AS1 was upregulated in GC samples. However, only HCP5 was induced by MSC co-culture in GC cells, indicating that MSC might regulate HCP5 to affect GC cells. Previous studies have revealed that MSCs released TGF-β-containing exosomes to transcriptionally induce lncRNA MACC1-AS1 in GC cells^32^. Therefore, it is also possible that HCP5 could be induced by certain exosome-derived factors from MSCs. It is also possible that HCP5 is an exosomal lncRNA transferred from MSCs to GC cells. However, the precise mechanism whereby MSCs induced HCP5 expression in GC cells was elusive in this study, and will be further explored in the future. Previously, HCP5 has been reported to promote cell proliferation and metastasis in multiple cancers, such as thyroid cancer, lung cancer, and colorectal cancer^41–43^. However, this was the first time for HCP5 to be explored in GC and related to drug-resistance. Functionally, we demonstrated that overexpression of HCP5 conferred chemo-resistance and improved stemness of GC cells.

Mechanistically, interaction of lncRNAs with miRNAs in cancer progression and cellular response to chemo-therapy has been widely documented^32,36^. Herein, we first identified that miR-3619-5p was a target for HCP5 which could be downregulated by MSC co-culture in GC cells. Formerly, miR-3619-5p has been depicted as a tumor-suppressor that repressed tumorigenesis and cell growth in prostate cancer, lung cancer and bladder cancer^63–65^. However, its relation with GC and chemo-resistance was first uncovered by our study. We validated that miR-3619-5p interacted with HCP5 and could counteract the facilitative effect of HCP5 on stemness and chemo-resistance of GC cells, indicating that miR-3619-5p served as a chemo-sensitizer in GC cells and HCP5 regulated chemo-resistance of GC cells through miR-3619-5p.

Furthermore, KEGG analysis through bioinformatics tool suggested that miR-3619-5p was closely related to FAO and AMPK pathways. It has been reported that facilitation of FAO contributed to the resistance of cancer cells to drugs and aggravated tumorigenesis^25–30^. Previous works have associated FAO to GC by demonstrating that FAO induced the metastasis and that MSC co-culture aggravated FAO and improved stemness and chemo-resistance in GC^31,32^. Therefore, it was reasonable to deduce that HCP5/miR-3619-5p could regulate FAO in GC. Unsurprisingly, we confirmed that HCP5 could increase FAO level through inhibiting miR-3619-5p.

Then, we explored the detailed mechanism whereby HCP5/miR-3619-5p regulated FAO. AMPK pathway has been proved by several reports to exert regulatory impacts on FAO metabolism^51–54^. Herein, we first identified that miR-3619-5p targeted PPARGC1A, an important factor in AMPK pathway. PPARGC1A encodes the protein named PGC1α, which is known as the transcriptional co‐activator modulating FAO^55^. Furthermore, a previous literature stated that CPT1, a major regulator of FAO, could be transcriptionally activated by PGC1α/CEBPB complex, so that the FAO in cancer cells was facilitated^56^. Herein, we first revealed that HCP5 increased the expression of PPARGC1A to promote the production of PGC1α and the formation of PGC1α/CEBPB complex, leading to transactivation of CPT1 in GC cells. Finally, we validated through rescue assays that HCP5 enhanced stemness and chemo-resistance in GC through PPARGC1A-mediated FAO. In vivo assays showed that HCP5 could abrogate the anti-tumor effect of FOLFOX regime, indicating that targeting HCP5 could be an innovative way to higher the efficacy of chemotherapy in GC.

## Conclusions

**Fig. 8:**
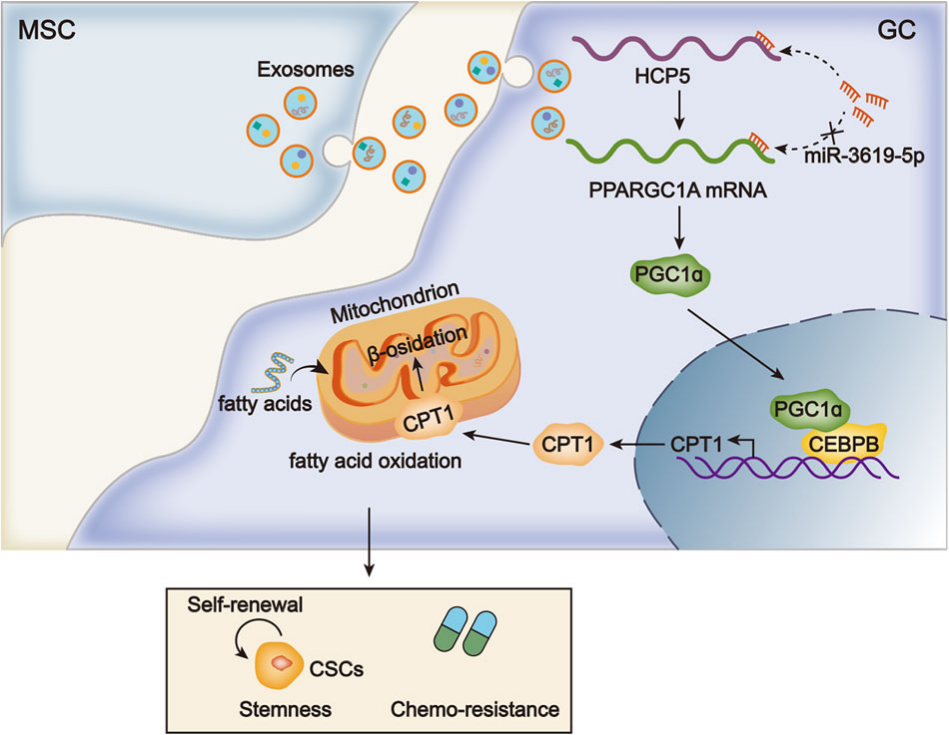
Schedule diagram of the findings in this work. MSCs induced HCP5 level in GC cells. HCP5 sponged miR-3619-5p to induce PGC1α and facilitate CEPBP/PGC1α complex-mediated transactivation of CPT1 so as to aggravate FAO, stemness, and chemo-resistance in GC cells.

This study first revealed that lncRNA HCP5 was induced by MSC in GC cells. Functionally, we confirmed that HCP5 conferred chemo-resistance and enhanced stemness of GC cells. Mechanistically, we demonstrated that HCP5 targeted miR-3619-5p to upregulate PPARGC1A, leading to the transactivation of CPT1 by PGC1α/CEBPB complex and facilitated FAO (Fig. 8). These findings suggested that HCP5 could be a promising target for the improvement of chemotherapy efficacy in GC.

## Supplementary information

Supplementary Figure 1

Supplementary Figure 2

Supplementary Figure 3

Supplementary Figure 4

Supplementary figure legends

Supplementary table 1
