# Supplementary figures and images for: MSC-induced lncRNA HCP5 drove fatty acid oxidation through miR-3619-5p/AMPK/PGC1α/CEBPB axis to promote stemness and chemo-resistance of gastric cancer
Source: Cell Death Dis. 2020 Apr 16;11(4):233. doi: 10.1038/s41419-020-2426-z (PMC7162922; doi:10.1038/s41419-020-2426-z)

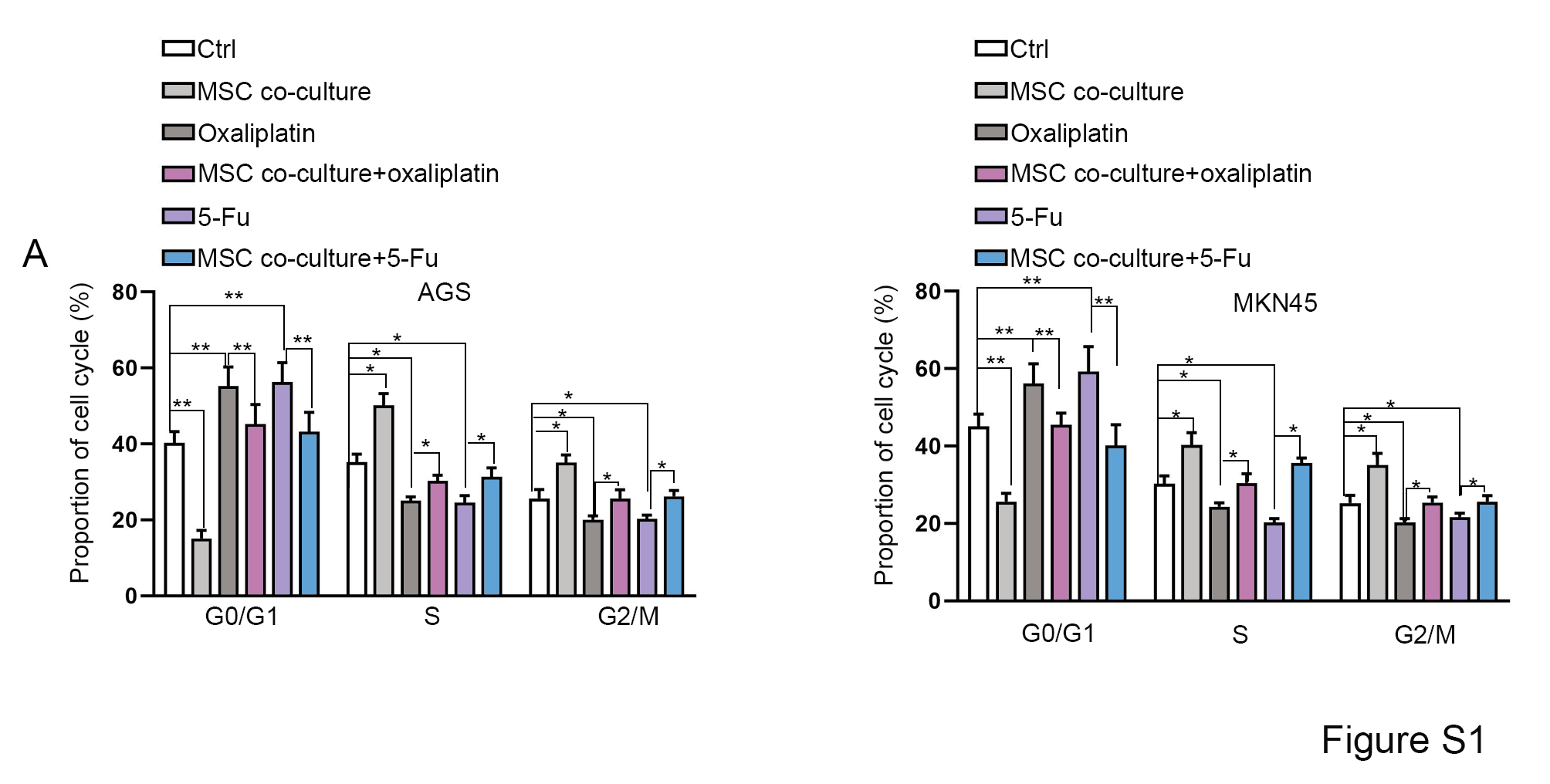

Supplement: Supplementary file 1 — Supplementary Figure 1 [file 41419_2020_2426_MOESM1_ESM.tif]

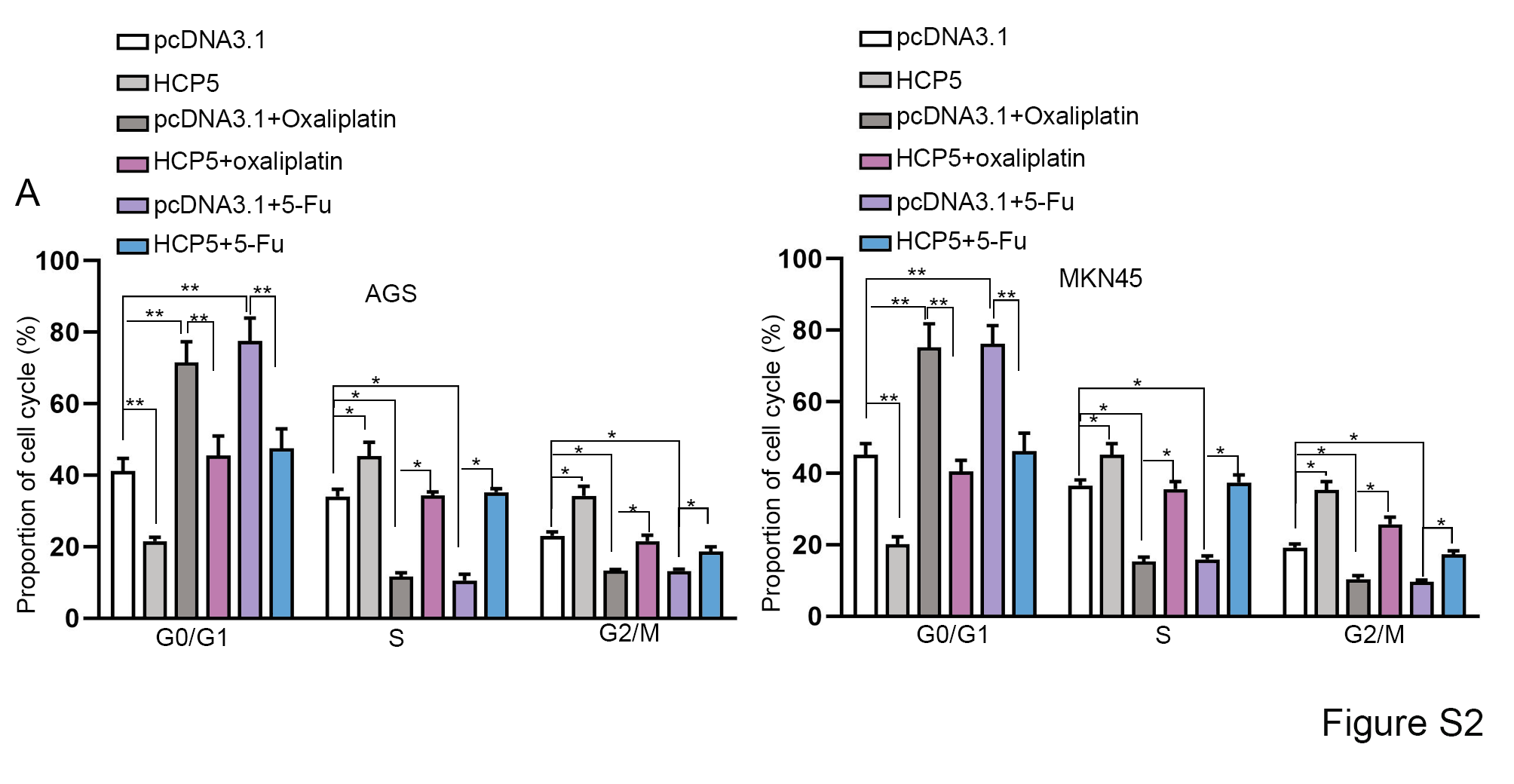

Supplement: Supplementary file 2 — Supplementary Figure 2 [file 41419_2020_2426_MOESM2_ESM.tif]

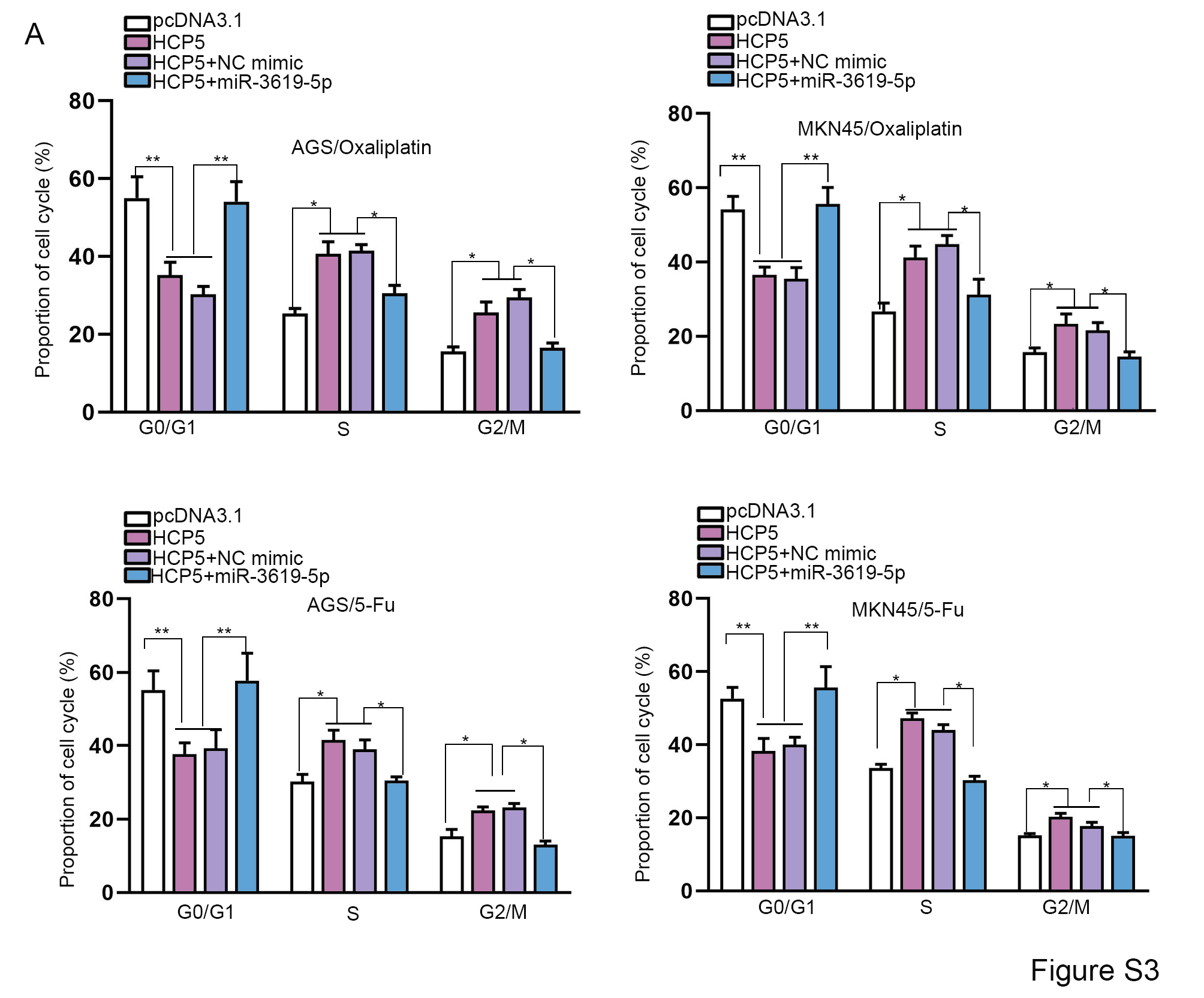

Supplement: Supplementary file 3 — Supplementary Figure 3 [file 41419_2020_2426_MOESM3_ESM.tif]

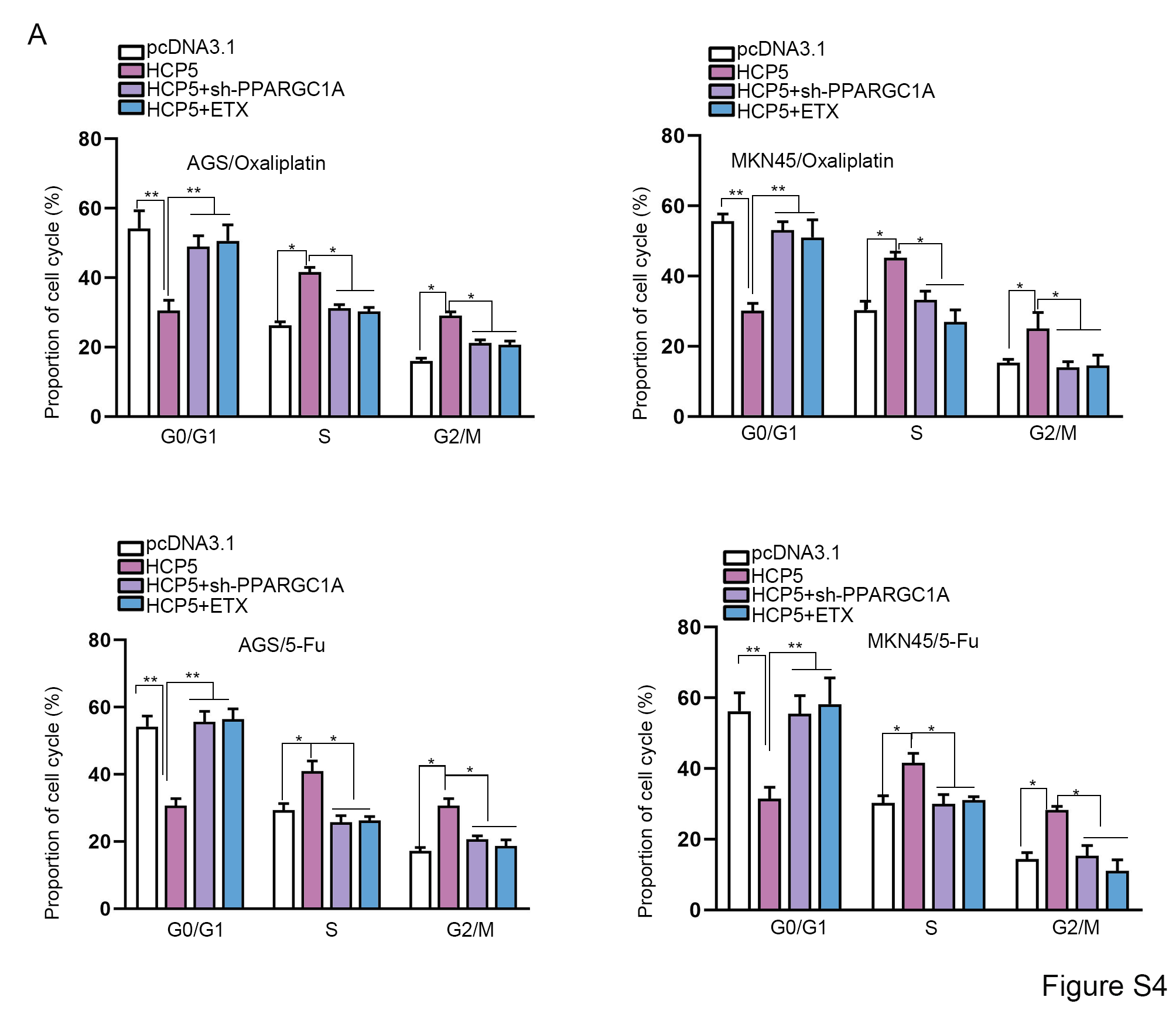

Supplement: Supplementary file 4 — Supplementary Figure 4 [file 41419_2020_2426_MOESM4_ESM.tif]
