# Supplementary material for: MSC-induced lncRNA HCP5 drove fatty acid oxidation through miR-3619-5p/AMPK/PGC1α/CEBPB axis to promote stemness and chemo-resistance of gastric cancer
Source: Cell Death Dis. 2020 Apr 16;11(4):233. doi: 10.1038/s41419-020-2426-z (PMC7162922; doi:10.1038/s41419-020-2426-z)
Supplement: Supplementary file 5 — Supplementary figure legends [file 41419_2020_2426_MOESM5_ESM.docx]

**Supplementary figure legends**

**Figure S1. Cell cycle analysis by flow cytometry. A.** GC cells were divided into 6 groups: Ctrl, MSC co-culture, Oxaliplatin, MSC co-culture+oxaliplatin, 5-Fu, or MSC co-culture+5-Fu. Ratio of cells at G0/G1, S, and G2/M phase was analyzed by flow cytometry. ^*^P < 0.05, ^**^P < 0.01. Error bars indicate SD. Each assay was conducted for 3 times.

**Figure S2. Cell cycle analysis by flow cytometry. A.** GC cells were divided into 6 groups: pcDNA3.1, HPC5, pcDNA3.1+Oxaliplatin, HPC5+oxaliplatin, pcDNA3.1+5-Fu, or MSC HPC5+5-Fu. Ratio of cells at G0/G1, S, and G2/M phase was analyzed by flow cytometry. ^*^P < 0.05, ^**^P < 0.01. Error bars indicate SD. Each assay was conducted for 3 times.

**Figure S3. Cell cycle analysis by flow cytometry. A.** GC cells were divided into 4 groups: pcDNA3.1, HPC5, HPC5+NC mimics, or HPC5 miR-3619-5p. Ratio of cells at G0/G1, S, and G2/M phase was analyzed by flow cytometry. ^*^P < 0.05, ^**^P < 0.01. Error bars indicate SD. Each assay was conducted for 3 times.

**Figure S4. Cell cycle analysis by flow cytometry. A.** GC cells were divided into 4 groups: pcDNA3.1, HPC5, HPC5+sh-PPARGC1A, or HPC5 +ETX. Ratio of cells at G0/G1, S, and G2/M phase was analyzed by flow cytometry. ^*^P < 0.05, ^**^P < 0.01. Error bars indicate SD. Each assay was conducted for 3 times.
